# Design and Benchmark Testing for Open Architecture Reconfigurable Mobile Spirometer and Exhaled Breath Monitor with GPS and Data Telemetry

**DOI:** 10.3390/diagnostics9030100

**Published:** 2019-08-21

**Authors:** Alexander G. Fung, Laren D. Tan, Theresa N. Duong, Michael Schivo, Leslie Littlefield, Jean Pierre Delplanque, Cristina E. Davis, Nicholas J. Kenyon

**Affiliations:** 1 Department of Mechanical and Aerospace Engineering, University of California, Davis, CA 95616, USA; 2 Department of Internal Medicine, 4150 V Street, Suite 3400, University of California, Davis, Sacramento, CA 95817, USA; 3 VA Northern California Health Care System, 10535 Hospital Way, Mather, CA 95655, USA; 4 Center for Comparative Respiratory Biology and Medicine, University of California, Davis, CA 95616, USA

**Keywords:** breath analysis, spirometry, personalized medicine, telehealth

## Abstract

Portable and wearable medical instruments are poised to play an increasingly important role in health monitoring. Mobile spirometers are available commercially, and are used to monitor patients with advanced lung disease. However, these commercial monitors have a fixed product architecture determined by the manufacturer, and researchers cannot easily experiment with new configurations or add additional novel sensors over time. Spirometry combined with exhaled breath metabolite monitoring has the potential to transform healthcare and improve clinical management strategies. This research provides an updated design and benchmark testing for a flexible, portable, open access architecture to measure lung function, using common Arduino/Android microcontroller technologies. To demonstrate the feasibility and the proof-of-concept of this easily-adaptable platform technology, we had 43 subjects (healthy, and those with lung diseases) perform three spirometry maneuvers using our reconfigurable device and an office-based commercial spirometer. We found that our system compared favorably with the traditional spirometer, with high accuracy and agreement for forced expiratory volume in 1 s (FEV1) and forced vital capacity (FVC), and gas measurements were feasible. This provides an adaptable/reconfigurable open access “personalized medicine” platform for researchers and patients, and new chemical sensors and other modular instrumentation can extend the flexibility of the device in the future.

## 1. Introduction

The benefits of home and personal spirometry have been established in a variety of lung diseases, including chronic obstructive pulmonary disease (COPD), severe asthma, pulmonary fibrosis and for patients post lung transplant [1,2].

Home spirometers are available in different styles, and they are grossly classified as volumetric (i.e., wet and dry spirometers) or flow measuring (i.e., pneumograph systems, mass flow meters). Volumetric spirometers directly measure inhaled or exhaled volume, and flow spirometers measure the speed at which the air flows. The volumetric spirometers are smaller, and more likely to be used at home. The shape and size of commercial devices vary, from a smartphone handheld device to a table-top instrument, with a laptop sending immediate results electronically to the physician. However, there are no wearable and reconfigurable devices available to either the research community or patients.

Asthma is just one disease where home spirometry can be of use. It is a chronic inflammatory disease of the airways that is characterized by airway hyper-reactivity and episodic attacks upon exposure to various inhaled aeroallergens and other environmental stimuli. Approximately 300 million people worldwide are afflicted with asthma [3], with twenty-five million asthmatics residing in the United States alone [4]. Treatments often need to be initiated at once to prevent increased risks related to asthma attacks, including emergency room visits, hospitalizations, and even death [5]. Asthma and COPD are of particular interest because they exemplify two diseases where the combination of lung spirometry with exhaled breath metabolite testing may be of value, and instruments that can be adapted for specific use are very valuable.

Exhaled asthma breath biomarker research is a relatively new area that has the potential to transform healthcare in the US and improve present management strategies. Indeed, we now have supporting evidence that both volatile and non-volatile compounds in patient breath can be monitored and associated with disease status [6]. Thousands of chemicals and small proteins have been detected in breath using a variety of large bench-top instrumentation platforms and sensors. Asthma is a disease across the lifespan. Young populations are usually early adopters of new technologies, and they are very likely to embrace and use new miniature hand-held tools to help monitor their own health if it helps them feel better on a daily basis. But for children to carry around and adopt these new devices, they need to be drastically miniaturized and compatible in size with other technology (e.g., cell phones, inhalers).

We have previously developed a novel portable technology to measure lung function and exhaled breath gases. This tool has been expanded as a modular and highly reconfigurable design for use as a research tool. We tested our device in patients with COPD and asthma, along with healthy control patients. Our specific objective was to combine commercial-off-the-shelf electrochemical sensors for nitric oxide (NO), and carbon monoxide (CO) within the device to measure these compounds in exhaled breath. To this end, we developed an open-architecture device that combines spirometry (FEV1, FVC, and spirometry graph), and chemical breath biomarker measurements of NO and CO. Ultimately, our breath analysis technology could be instrumental in establishing the first open architecture “personalized medicine” platform for asthmatic adults and children. By enabling the design with commercial-off-the-shelf parts, open access microcontroller code and open access software, we allow this tool to be brought into mainstream use for continued development by other researchers.

## 2. Materials and Methods

### 2.1. Open Access Reconfigurable Spirometer with Breath Gas Sensors

**Figure 1:**
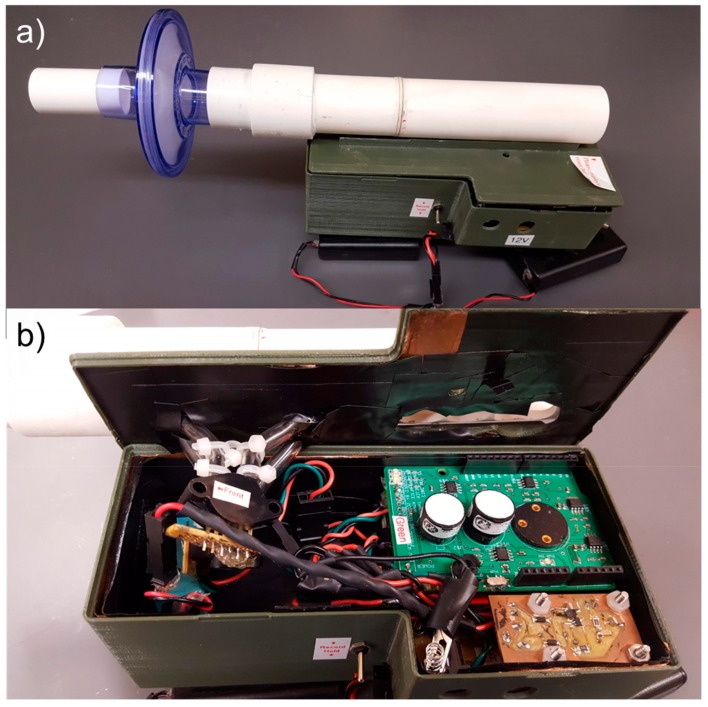
An open access platform is provided for a portable reconfigurable pulmonary lung function measurement system. It interfaces with common commercial android-based personal mobile devices for seamless real-time data monitoring and data telemetry. Commercial microcontrollers and custom printed circuit boards (PCBs) provide a modular approach to the platform design. Additional sensors can be integrated into this device over time. (**a**) Enclosed device. (**b**) Device interior with stacked PCBs and flow sensor.

The base engineering design of our novel device was previously published [7,8]. In this current report, we have updated the operating systems, updated and provided the software/code that controls the device, and we provide all of the printed circuit board (PCB) designs required to make a reconfigurable device that is fully open access for non-profit academic research use. An image of the device is provided (Figure 1).

The overall architecture of the device includes piezoresistive pressure sensors selected to monitor across a broad range of high flows of 50–900 L/min (pressure sensor A; model #MPX5010; Freescale Semiconductor; San Jose, CA, USA), and low flows of 15–100 L/min (pressure sensor B; model #SSCSNBN002NDAA5; Honeywell; Morristown, NJ, USA). Two commercial off-the-shelf (COTS) electrochemical, chemical sensors (NO, CO) were integrated into our system (model numbers NO-D4 and CO-D4; AlphaSense Ltd.; Essex, UK). To correctly operate the NO and CO sensors, a potentiostatic circuit was built to control the chemical sensor (Supplemental Figure S1), and a transimpedance amplifier (Supplemental Figure S2) was used to convert the current generated from the NO and CO sensors to a measurable voltage. The GPS location for each spirometry maneuver was recorded using the mobile device’s GPS information. While our device is in use it is connected to the mobile device (Motorola Xoom, Android Operating System version 4.0.4), which captures the data in real time via an installed custom-designed software application (available on GitHub).

The system has been improved from the previous device [7] in several ways. The voltage supply to the chemical sensors was updated to include regulating circuitry in order to provide a constant voltage, and the coin cell batteries were replaced with rechargeable batteries. An on-off switch and a data collection switch were added to improve user operation. The interior of the circuitry case was coated with copper tape and grounded to minimize external noise. Additionally, the software was updated with improved timing.

### 2.2. Benchmark Testing of the Device

A cross-sectional observational study was conducted at the University of California Davis (UCD) pulmonary clinics. Subjects were recruited by advertisement and personally from the UCAN™ asthma network clinic, UCD pulmonary rehabilitation program, and through the pulmonary research office. Adult subjects who were healthy with no diagnosis of chronic obstructive pulmonary disease (COPD) or asthma, and those who had a physician diagnosis of either asthma or COPD, were recruited. All participating subjects gave their written informed consent and filled out a case report form that gathered demographic data. The subjects who were able to perform the required repetitive respiratory maneuvers were included. The study was approved by the University of California, Davis Institutional Review Board (IRB) Administration (487379-3).

### 2.3. Inclusion and Exclusion Criteria

The subject screening protocol identified patients who met the criteria for COPD, asthma, or healthy control subject groups. For COPD subjects, the patients had a physician diagnosis of COPD, were prescribed COPD medications, and were either an active smoker or had a greater than fifteen pack year history of smoking. The asthma subjects had a physician diagnosis of asthma, were prescribed an asthma controller medication, were not an active smoker, and had a less than ten pack year history of smoking. The healthy control subjects had neither a diagnosis of asthma nor COPD, were not active smokers, and had a less than ten pack year history of smoking. All subjects had to have the ability to provide informed consent and be willing to perform repeated forced vital capacity maneuvers. Patients who were pregnant or were under 18 years old were excluded. Patients who could not perform reliable consecutive respiratory maneuvers per established criteria [9,10] were excluded, patients with respiratory illness and/or in an acute COPD or asthma exacerbation were also excluded.

### 2.4. Testing of Novel Versus Conventional Spirometry

After providing informed consent, each subject underwent the following process: They filled out a demographic and data questionnaire and then performed respiratory maneuvers with the novel device, the conventional spirometer, and the standard nitric oxide device (NIOX MINO, Aerocrine Inc., Morrisville, NC, USA). Adult subjects were asked to perform three spirometry maneuvers under the supervision of a trained health care professional using the conventional clinical spirometer (SPIROLAB II, SDI Diagnostic, Easton, MA, USA). The same subjects were then asked to perform three more respiratory maneuvers using our novel device and the NIOX MINO. In brief, each subject was coached by a healthcare-trained professional to perform a few tidal breaths using the spirometer. When the subject was ready, a deep inhalation was followed quickly by a fast, forceful exhalation with the exhalation performed until completion, then the subject quickly deeply inhaled. This created a flow volume loop, and the subject was asked to repeat this until three flow volume loops were created.

Our sensors were calibrated to detect the lower end of the biomarker concentration range found in the exhaled breath of asthmatic patients (0.03 ppm NO, 2 ppm CO). Subjects had five seconds of rest before the device signals the patient to perform the spirometry maneuver. Without removing the spirometry device from the oral cavity, the subjects performed a deep inhalation followed by a quick, forceful exhalation maneuver and the subject is prompted to exhale for at least six seconds. Quantification of chemical biomarkers in exhaled breath must also occur before spirometry maneuvers, because the spirometry often causes exhaled NO concentrations to artificially decrease, thus skewing the actual concentration of NO [11].

### 2.5. Statistical Analysis

We compared the spirometry between healthy subjects, asthmatics, and COPD subjects for the conventional spirometry and the novel open access mobile spirometer. To understand the bivariate relationship between the novel device and the conventional spirometer in asthmatics for forced expiratory volume in 1 s (FEV1) and forced vital capacity (FVC), scattered plots were created. Two-factor Analysis of Variance (ANOVA) (device and health state) was performed in Matlab R2017A (Mathworks, Natick, MA, USA). The Pearson’s R Correlation tests were performed to compare these FEV1 and FVC values for the conventional spirometer and the novel device. This correlation analysis was performed across all subjects. Additionally, Bland-Altman plots were generated and interpreted.

## 3. Results

### 3.1. Subject Characteristics

In total, 49 subjects were enrolled to benchmark the device (Table 1), and all subjects were asked to perform three spirometry maneuvers using both the standard SPIROLAB spirometer and the novel device under the supervision of a health care professional. The same subjects were then asked to perform three more spirometry maneuvers using the novel device. Of the 49 subjects, 43 were able to perform three acceptable maneuvers on three attempts on the commercial spirometer. Acceptable maneuvers were evaluated in accordance with the requirements established by the American and European Respiratory Societies [10,12], where the two largest values of FEV1 and FVC must be within 0.150 L. Only forced exhalation is assessed with the novel device, thus only PEF, FEV1 and FVC are recorded. Prior to each novel spirometer maneuver, subjects were asked to perform tidal breaths for 60 sec, and during this period, exhaled nitric oxide and carbon levels were measured.

### 3.2. Benchmarking Spirometry

We found 43 of the 49 control, asthmatics and COPD subjects were able to perform three acceptable spirometry maneuvers on the clinical spirometer and three spirometry maneuvers on the novel spirometer, with a total of six total vital capacity maneuvers. Spirometry, in particular the expiratory limb of the flow volume loop, captures the flow rate, which increases at a steep positive slope until the peak flow is reached, approximately one second from the start of exhalation (FEV1).

**Figure 2:**
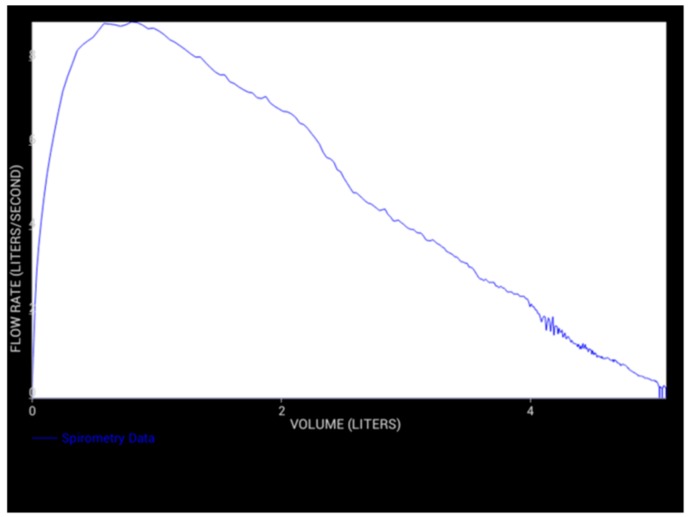
Typical exhalation limb of the flow-volume loop tracing produced by the novel spirometer. This figure is produced in real-time on the android-based interface. Additional sensors may be added to the device over time, and their outputs can also be visually monitored.

At the end of the six seconds of exhalation, the flow rate reaches zero, and the corresponding x-axis value would be considered the total lung capacity for that subject (Figure 2).

**Figure 3:**
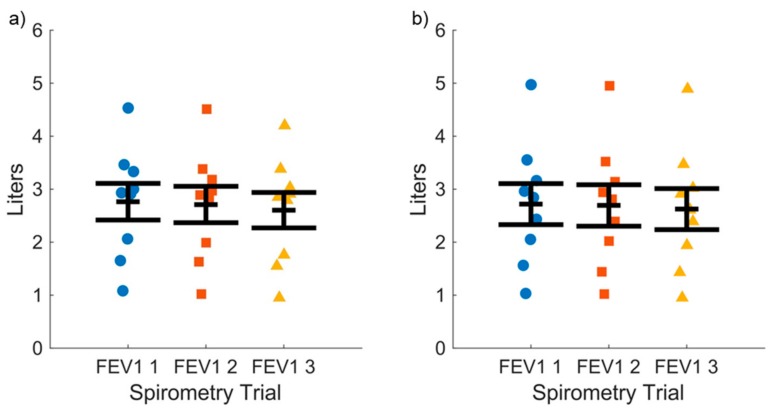
Forced expiratory volume in 1 s (FEV1) values measured across three consecutive vital capacity maneuvers for asthma subjects for: (**a**) Spirometer function of our novel platform; and (**b**) a commercial bench-top traditional spirometer. Mean ± SEM values for the novel and traditional spirometer are shown.

**Figure 4:**
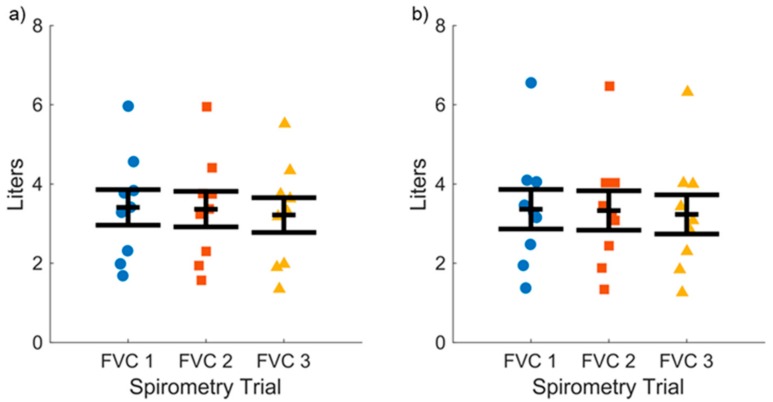
Forced vital capacity (FVC) values measured across three consecutive vital capacity maneuvers for asthma subjects for: (**a**) Our open access modular spirometer; and (**b**) the traditional commercial spirometer. Mean ± SEM values for the novel and traditional spirometer are shown.

Accuracy of the novel device compared to the conventional spirometer was assessed by the FEV1 and FVC values. The mean and SEM values for three successive FEV1 maneuvers for asthmatic subjects (*n* = 17) are shown for the novel (Figure 3a) and clinical spirometer (Figure 3b), respectively, which demonstrate the subject variability in the data. The mean ± SEM values for FEV1 for all subjects for the novel spirometer (2.35 ± 0.15 L) and conventional spirometer (2.37 ± 0.16 L) were not statistically different via two-factor (device and health state) ANOVA (F = 0.38, *p* = 0.538). The mean SEM values for three successive FVC maneuvers for asthmatic subjects (*n* = 17) are shown for the novel (Figure 4a) and conventional spirometer (Figure 4b), respectively. The mean ± SEM values for all subjects for FVC for the novel (3.05 ± 0.17 L) and conventional (3.02 ± 0.18 L) were not statistically different via two-factor (device and health state) ANOVA(F = 1.13, *p* = 0.29).

**Figure 5:**
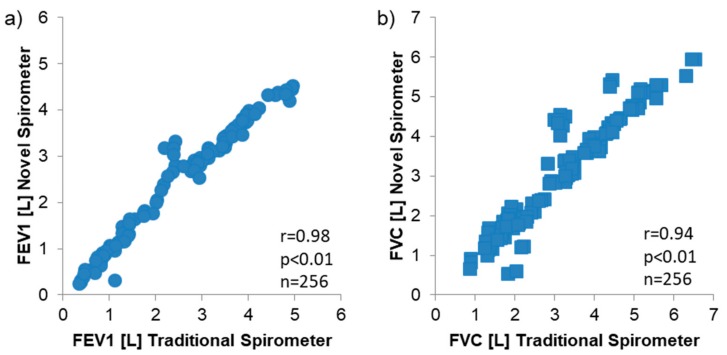
Correlation between values measured from the traditional and novel spirometer platform with *n* = 256 vital capacity maneuvers were performed on a total of 43 subjects with asthma, chronic obstructive pulmonary disease (COPD), and healthy controls, (**a**) FEV1, (**b**) FVC.

The correlation between spirometers for all tests (*n* = 256, two maneuvers not completed) was remarkable for FEV1 (R = 0.98, *p* < 0.01; Figure 5a) and FVC (R = 0.94, *p* < 0.01; Figure 5b).

**Figure 6:**
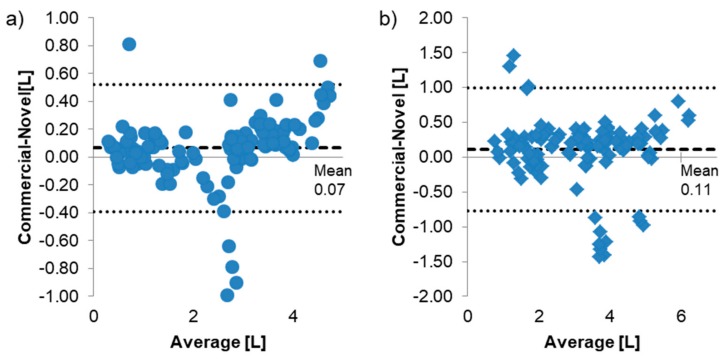
Bland-Altman Plots for (**a**) FEV1, (**b**) FVC. The dashed line indicates the mean and dotted lines indicate the 95% limits of agreement (mean ± 1.96 standard deviations).

Bland-Altman plots with 95% limits of agreement are shown in Figure 6. There is a slight bias for both FEV1 and FVC; however, this difference is within the bounds of repeated spirometry maneuvers (150 mL).

### 3.3. Exhaled Gas Measurements

The dynamic ranges for breath chemical biomarkers found in exhaled breath in asthma patients are 0.03–0.13 ppm for NO [15,16,17] and 2–7 ppm for CO [18,19]. Determining these ranges help define the testing protocols needed to ensure that the chemical sensors used in the novel device are capable of correctly measuring concentrations of NO and CO in a breath. Quantification of chemical biomarkers in exhaled breath must also occur before spirometry maneuvers, because the spirometry often causes exhaled NO concentrations to artificially decrease, thus skewing the actual concentration of NO [11].

All readings were within the “physiologic” ranges compared to known values in humans, despite the fact that we are not able to measure against a known gold standard exhaled CO device. Mean and SEM carbon monoxide readings for all *n* = 127 sample maneuvers was 3.04 ± 0.22 ppm. Intra-participant correlation coefficients between two sets of readings ranged from a low of 0.57 (*p* < 0.01 value 1 versus value 2) to a high of 0.72 (*p* < 0.01, value 1 versus value 3) for all readings tested.

In contrast, exhaled NO values were not as consistent using the specific commercial metal oxide sensor chosen with an average ± SEM reading of 153.78 ± 15.4 ppb (*n* = 127), compared to 24.33 ± 1.90 ppb for the commercial device (*n* = 84, one subject could not perform the maneuver). There was no significant correlation between the average commercial device readings and average metal oxide sensor readings (*p* = 0.28). We believe this was most likely a result of the electrochemical NO sensor utilized in our device, rather than an artifact of varying exhaled flow rates. However, with the open access architecture, it is possible for researchers to select various chemical sensors and incorporate them into the platform.

## 4. Discussion

In our current work, we provide all of the technical information, microcontroller code, software and PCB designed for an open-access reconfigurable platform, shown in Figure 1. The benchmarking study was designed to determine if an inexpensive, open access mobile platform could be optimized to perform pulmonary lung function tests and quantify relevant exhaled breath biomarkers (NO, CO in a cohort of patients with obstructive lung disease). We also wanted to test the platform accuracy and compare it to that of a commonly-used commercial benchtop spirometer.

We found that the novel device, when compared with the traditional spirometer, correlated extremely well for all subject groups. The accuracy of the repeated measurements for both FEV1 and FVC values were near-equal between the two spirometers. Bland-Altman plots showed a slight bias between the commercial and novel device. This bias could be from the experimental design where subjects first performed spirometry maneuvers on the commercial device prior to the novel device.

By using the novel spirometer second, subjects may have tired and not performed as well. Also, the two sets of spirometry maneuvers were treated independently from each other, so subjects were coached to perform their best on each device, and not to perform acceptable maneuvers across both devices.

Measurement of exhaled CO in end tidal breaths was feasible with values that were physiologically appropriate, while exhaled NO measurements were more variable. Overall, these findings support a new open access prototype technology using an Android platform and device.

A spirometry test is normally performed at a clinic or hospital under the supervision of trained professionals, given that it has strict guidelines for reproducibility and acceptance [12]. However, we show in our benchmark testing, that portable devices may have great clinical utility. Two of the most important parameters gathered from a spirometry test are the forced vital capacity (FVC), described as the volume delivered during expiration when made as forcefully and completely as possible, starting from full inspiration, and the forced expiratory volume in one second (FEV1), which is the volume delivered in the first second of the FVC maneuver [9,10,12]. Graphical depictions of flow-volume and flow-time curves help visualize lung function, and allow physicians to evaluate lung health. Currently, patients are encouraged to monitor their asthma at home using simple PEF devices; they have been shown to decrease the number of severe asthma episodes [20], but are ineffective when individuals fail to consistently adhere to these plans [21,22]. This noncompliance may be due to the time and discipline required to manually assess asthma symptoms over a long time frame [23].

Asthma was a good use test cohort for this technology. Asthma is particularly challenging for health care professionals to effectively manage due to the severity of symptoms, triggers and any responsiveness to treatment medications which are often unique to each individual. An inexpensive mobile open access portable monitoring device would provide the data necessary to formulate a personalized management plan and identify the variables that may lead to an asthma attack. A comprehensive guideline for an asthma action recommends focusing on monitoring asthma patients with a combination of symptoms, rescue drug use, and expiratory flows with a goal to provide timely and appropriate asthma therapy [24]. The surrogate, non-specific tests now available for asthma diagnosis and monitoring include spirometry, peak expiratory flow measurement, and a non-invasive marker of airway inflammation known as fractional exhaled nitric oxide (FeNO) [25,26].

Our clinical benchmarking data provides accuracy and precision estimates for a novel, reconfigurable, portable device, especially for patients with asthma and COPD. A novel exhaled breath monitoring device was developed that combines spirometry (FEV1, FVC, and flow-volume loop), and chemical breath biomarker measurements (nitric oxide and carbon monoxide) into two breath maneuvers. A custom software application for Android mobile technology was developed to interpret, display, record and email the data to a health care professional for personalized care. By coupling the novel exhaled breath monitor with Android mobile technology through a universal serial bus (USB), patients will be able to monitor their asthma symptoms from any setting, and be encouraged to adhere to their prescribed asthma monitoring plan. Initial testing of the device in the laboratory setting demonstrated that it was capable of measuring primary lung function parameters (FEV1, FVC) with reasonable accuracy and precision [7]. The flow determination uses a fixed airflow obstruction design equipped with two differential pressure sensors, each measuring one section of the expected volume flow rate range. Despite what may seem as a limitation and a barrier to measurement at these low flows, the flow volume loops visually appeared acceptable with a slope that gradually approached zero. Pearson correlation for the FVC values (R = 0.94) was very strong, and the accuracy of the novel spirometer compared to traditional and clinically-available spirometer for all three groups was extremely high, and validates the device in this limited cohort of subjects.

For this modular custom designed prototype, commercially-available and inexpensive electrochemical sensors were selected for their combination of cost, high sensitivity, miniature size and short response time compared to other available chemical sensors. We have previously shown that they have a linear relationship between voltage and concentration of analyte, and each chemical sensor was able to detect various concentrations of its target analyte accurately. End tidal breathing measurements for exhaled carbon monoxide (CO) were very reliable.

Although our values were not measured for precision against a gold standard exhaled CO monitor, our findings were in the physiologic ranges compared to known values in non-smoking humans, and were reliable across three maneuvers. The mean ± SEM CO readings for all *n* = 127 samples was 3.04 ± 0.22 ppm. Correlation coefficients between readings ranged from 0.57 to 0.72 for all values tested. When testing in these subject groups, and in contrast to the spirometry measurements, measurements of exhaled NO (eNO) values were not always reliable. Variable equilibration time of these particular electrochemical sensors may have also contributed to our results. Testing with better electrochemical sensors is warranted.

The modular design allows for other sensors to be combined with the device. As research into exhaled breath grows, additional breath biomarkers are likely to be discovered. Sensors for these biomarkers could be added in the future.

Proper maintenance of the device is critical to ensure the accuracy of the results. Daily calibration checks for the spirometer may be performed per recommendations [10], although this would be difficult for an at home patient. Instead, periodic checks and re-calibrations could be performed at the lab or office if the regularly collected data begins to deviate noticeably for the patient, despite no changes in health. Initially, the chemical sensors would intermittently need to be taken to a lab for validation and recalibration. Depending on the chemical, at home validation and/or calibration kits could be developed.

In this current work, we provide a platform architecture for future open access pulmonary lung function instrumentation development. By using commercial component parts and simple easy-programmed microcontrollers, we can couple this platform to simultaneously acquire data on a smart phone or tablet and utilize the built-in connectivity of the internet. The ability for portable and/or wearable sensors to gather physiological data quickly and efficiently, and then instantly communicate that data with a health care professional means that such devices have the potential to significantly improve the speed of respiratory health care, and also to improve how asthma and COPD is managed in the future [27].

## 5. Conclusions

We have designed and validated an open access lung function monitoring device that utilizes tablet technology to create a convenient, reliable and user-friendly system. Initial validation testing has demonstrated that measurements taken with this device are comparable to those of a standard benchtop-sized commercial spirometer used in outpatient clinics and in research studies, and satisfy the minimum requirements for spirometry as outlined by Miller and colleagues. We believe the open access nature of our platform will further move this device into the personalized medicine realm, and allow researchers to reconfigure the device with new sensors, as needed.

**Non-Profit Open Access Information**

The PCB design specifications, microcontroller code and software code for the reconfigurable spirometer device are available on GitHub. Please refer to Professor Cristina Davis’ webpage for more information. This material is available as open source for research and personal use under a Creative Commons Attribution-Non-Commercial 4.0 International Public License (https://creativecommons.org/licenses/by-nc/4.0/). Commercial licensing may be available, and a license fee may be required. All code and PCB designs are ©The Regents of the University of California, Davis campus, all other rights reserved. Future published scientific manuscripts or reports using this software and/or hardware designs must cite the original publication (DOI: 10.1109/JSEN.2014.2373134) and this updated configuration report (DOI: 10.3390/diagnostics9030100).

## Figures and Tables

**Table 1:** Demographics of clinical cohort and testing outcomes.

Characteristic	Control	Asthma	COPD
Age-Year *	37.73 ± 14.04	51.24 ± 16.00	69.91 ± 7.93
Male sex—% (No.)	67 (9)	35(6)	45 (5)
Height (cm) *	171.53 ± 9.35	162.71± 7.15	167.41 ± 16.08
Baseline Spirometry			
FEV1 (L) ᵠ	3.74 ± 0.54	2.37 ± 1.10	1.06 ± 0.55
FVC (L) ᵠ	4.42 ± 0.66	3.02 ± 1.24	2.05 ± 0.70
% Predicted FEV1 *	106.69 ± 12.32	80.77 ± 26.51	43.28 ± 23.95
% Predicted FVC *	104.20 ± 11.59	84.65 ± 22.05	64.62 ± 19.07
Race or Ethnic Group—% (No.)			
African-American	-	-	9 (1)
White	14 (2)	53 (9)	73 (8)
Other	86 (12)	47 (8)	18 (2)
Asthma Control Test *	-	18.2 ± 5.6	-
Smoking (Pack Years) *	-	-	41.4 ± 20.1

* Values are given as the mean ± SD. Predicted values calculated by recommended equations [13,14]. ᵠ Values are given as the mean of 3 performed maneuvers ± SD.

## Supplementary Materials

The following are available online at https://www.mdpi.com/2075-4418/9/3/100/s1, Figure S1: Nitric Oxide Sensor Circuit, Figure S2: Carbon Monoxide Sensor Circuit.
