# Supplementary figures and images for: Design and Benchmark Testing for Open Architecture Reconfigurable Mobile Spirometer and Exhaled Breath Monitor with GPS and Data Telemetry
Source: Diagnostics (Basel). 2019 Aug 21;9(3):100. doi: 10.3390/diagnostics9030100 (PMC6787596; doi:10.3390/diagnostics9030100)

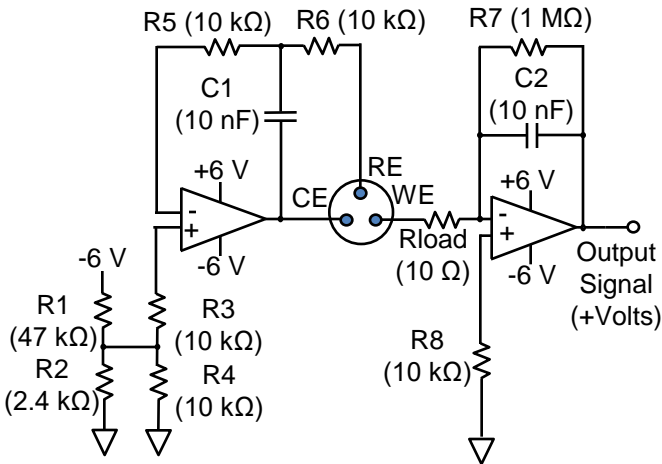

Supplement: Supplementary file 1 [file diagnostics-09-00100-s001.zip › Supplemental Files/Supplemental Figure 1.pdf]

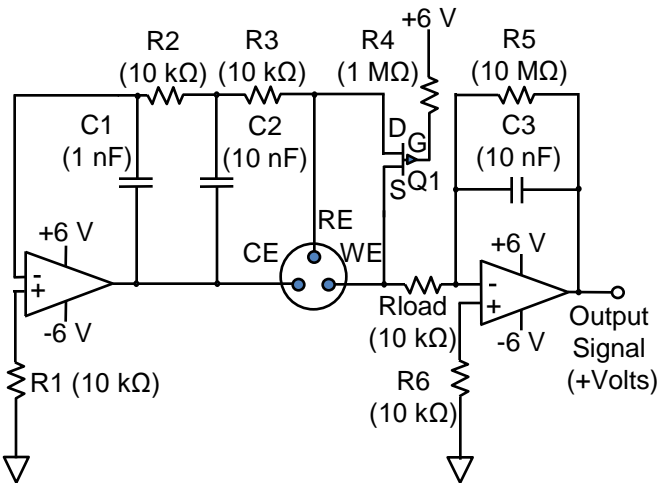

Supplement: Supplementary file 1 [file diagnostics-09-00100-s001.zip › Supplemental Files/Supplemental Figure 2.pdf]
